# First Polish outbreak of *Clostridium difficile* ribotype 027 infections among dialysis patients

**DOI:** 10.1007/s10096-014-2204-x

**Published:** 2014-07-26

**Authors:** D. Lachowicz, G. Szulencka, P. Obuch-Woszczatyński, A. van Belkum, H. Pituch

**Affiliations:** 1 Department of Medical Microbiology, Medical University of Warsaw, 5 Chałubiński Street, 02-004 Warsaw, Poland; 2 Department of Medical Microbiology, Provincial Hospital, Włocławek, Poland; 3 bioMérieux R&D Microbiology, La Balme Les Grottes, France

## Abstract

This report describes an outbreak of *Clostridium difficile* infection (CDI) in a nephrology ward in 2012, caused by the fluoroquinolone- and clindamycin-resistant polymerase chain reaction (PCR) ribotype 027 strains. An increase in the number of cases of diarrhoea was noted among patients hospitalised between 26 November 2012 and 17 December 2012 in a hospital in North Poland. Eight patients were on haemodialysis in the outpatient dialysis facility, while one patient was receiving peritoneal dialysis. The 027 strain could be detected in eight haemodialysis patients. One strain, isolated from the patient receiving peritoneal dialysis, belonged to PCR ribotype 001. In this study, we documented the first outbreak of CDI caused by a fluoroquinolone-resistant (FQR) *C. difficile* PCR ribotype 027 strain in Polish dialysis patients.

## Introduction

*Clostridium difficile* infection (CDI) is the most common cause of infections in healthcare facilities. The clinical manifestations of CDI range from asymptomatic colonisation to toxic megacolon, which has a high risk of mortality [1]. The three main risk factors for CDI include antibiotic exposure, higher age (65 years and above) and prolonged hospitalisation [2, 3]. Additional risk factors include cancer, haematopoietic stem cell transplant (HSCT), co-morbidities associated with immunosuppression, use of gastric acid-reducing drugs, admission to the intensive care unit, use of nasogastric tubes and inflammatory bowel diseases [4–7]. Patients with kidney disease are also at an increased risk of developing CDI and represent an especially vulnerable group of patients [8–10].

*C. difficile* NAP1/BI/027 strains have been reported to be aetiological agents of severe infections in the USA, Canada and Europe [11]. Recently, fluoroquinolones (FQ) have been linked to CDI, particularly those caused by polymerase chain reaction (PCR) ribotype 027 [12]. In the present study, we describe the epidemiology of CDI as well as the clinical and microbiological data of dialysis patients hospitalised in the nephrology ward of a hospital in Poland, with diarrhoea, caused by the PCR ribotype 027 strain.

## Materials and methods

### Patients

The current study was retrospective in nature, using *C. difficile* isolated from stool samples that were collected and stored when an outbreak was suspected in the nephrology ward. Diagnostic testing of CDI in the surveyed ward was performed only upon the physician’s request. The study was conducted at the 620-bed Provincial Hospital (PH), located in North Poland. In 2012, the incidence of CDI at the hospital was 4.5/10,000 patient-days (results not shown). Between 1 January 2012 and 31 December 2012, CDI was suspected in 96 symptomatic patients. Of these, 53 tested positive for *C. difficile* toxins A/B. The CDI-positive patients were hospitalised in different wards. Between 26 November 2012 and 17 December 2012, a high incidence of diarrhoea was observed in the 32-bed nephrology unit. These cases included nine dialysis patients with diarrhoea. Our study focused only on patients hospitalised in the nephrology ward who were receiving dialysis in the outpatient haemodialysis facility.

### Definitions

Diarrhoea was defined on the basis of well-known criteria [13, 14]. According to the European Centre for Disease Prevention and Control (ECDC), CDI can be classified into two types: healthcare-associated CDI, which usually occurs in a hospital after 48 h of admission, and community-associated CDI [15]. A severe case of CDI is defined by fever (>38.5 °C), decreased kidney function, high leukocyte count (>15 × 10^9^ cells/L) or colonic wall thickening revealed by a computed tomography (CT) scan [15]. Recurrent CDI is diagnosed when CDI re-occurs within 8 weeks of the onset of a previous episode, provided the symptoms from the previous episode resolved after the completion of initial treatment. An outbreak of CDI is defined as two or more cases related in time and place over a defined period (28 days), based on the date of onset of the first case. In this study, a possible CDI outbreak was identified based on the occurrence of three cases within 30 days, a departure from the normal pattern [16].

### Collection of demographic and clinical data

Medical records of the patients were studied for the presence of clinical symptoms of CDI. Data, including information on patient demographics, age, sex, admission type, diagnoses, length of stay, type of dialysis, use of antibiotics, type of discharge, recurrences and mortality, were obtained for 26 November 2012 through 17 December 2012. Stool character and frequency, and abdominal pain were also monitored. The characterisation of dialysis patients with diarrhoea due to *C. difficile* is shown in Table 1.

**Table 1:** Characterisation of dialysis patients with diarrhoea due to *Clostridium difficile* strain polymerase chain reaction (PCR) ribotype 027 and a sporadic case due to PCR ribotype 001

No./sex/age (years)	Date of hospitalisation D/Mo/Yr	PCR ribotype	Type of dialysis	AB	Date of onset of CDI	Leucocytosis ×10^9^ L	1.5× increase in CL	Recurrence	Fatality associated with CDI
1/F/52	15/11–31/12/2012	027	HD (one episode)	AMC, II° cephalosporin, II° fluoroquinolone, fluconazole	26/11/2012	15.67	Yes	No	No
2/F/61	14/11–01/12/2012	027	HD	AMC	28/11/2012	22.21	Yes	No	No
3/M/61	15/11–31/12/2012	027	HD	Aminopenicillin (amoxicillin, ampicillin)	30/11/2012	15.9	Yes	No	No
4/F/77	02/12–17/12/2012	027	HD	VA (iv), gentamicin	03/12/2012	17.38	No	Yes	Yes
5/M/73	19/11–17/12/2012	027	PD	I° and II° cephalosporin	04/12/2012	10.11	Yes	Yes	No
6/M/85	01/12–24/12/2012	027	HD	AMC, II° cephalosporin, macrolide	04/12/2012	17.5	No	No	No
7/F/77	05/12–27/12/2012	027	HD	II° cephalosporin, aminoglycoside, II° fluoroquinolone	05/12/2012	17.8	Yes	Yes	Yes
8/F/89	07/12–24/12/2012	027	HD	IP	11/12/2012	15.62	Yes	No	No
9/M/72	01/12/2012–04/01/2013	001	HD	Fluconazole	06/12/2012	3.12	No	No	No

*AB* antibiotics, *CDI C. difficile* infection, *CL* creatinine level, *HD* haemodialysis, *PD* peritoneal dialysis, *AMC* amoxicillin and clavulanic acid, *I°* first generation, *II°* second generation, *VA* vancomycin, *iv* intravenous, *IP* imipenem

### Microbiological methods

#### Detection of *C. difficile* toxin and culture of *C. difficile* strains

CDI in patients who developed diarrhoea was diagnosed by an immune-enzymatic assay for glutamate dehydrogenase (GDH; TechLab®, Inc., USA). In samples that tested positive in the GDH assay, CDI was confirmed using the *C. difficile* TOX A/B Test II™ kit (TechLab®, Inc., USA) and the detection of toxigenic *C. difficile* in culture. *C. difficile* was cultured from faecal samples using selective CLO Agar (bioMérieux SA; Marcy l’Etoile, France). After incubation at 35 °C for 48 h under anaerobic conditions, isolates were confirmed to be *C. difficile* on the basis of well-known criteria. All *C. difficile* strains were frozen at −70 °C in a Microbank™ bacterial storage system (Pro-Lab Diagnostics, UK). *C. difficile* isolates from the dialysis patients were collected for further analysis.

#### Real-time (RT)-PCR and PCR ribotyping

All *C. difficile* strains were sent to the Anaerobe Laboratory (AL) in Warsaw and tested for genetic markers of toxigenicity using the Xpert CD assay (Cepheid; Sunnyvale, CA, USA). The Xpert CD assay can be used to detect toxigenic *C. difficile* strains and identify the *C. difficile* NAP1/BI/027 strain. RT-PCR was performed to detect the *tcdB*, *cdtA* and *cdtB* genes (binary toxin genes), and a deletion in the *tcdC* gene (encodes a negative regulator of toxin A/B production). PCR ribotyping was performed as described by Stubbs et al. [17]. Binary toxin-producing control strains belonging to the Cardiff-ECDC reference strain collection were provided by Jon Brazier (Anaerobe Reference Laboratory, Cardiff, UK) and Ed Kuijper (Leiden University Medical Center, Leiden, The Netherlands), and consisted of R8637 (PCR ribotype 019), R5989 (PCR ribotype 023), R10456 (PCR ribotype 056) and strains from PCR ribotypes 045, 078, 027 and 176. The banding patterns obtained for the *C. difficile* strains were compared with those obtained for the reference strains.

#### Antimicrobial susceptibility testing

*C. difficile* strains were grown on Columbia Agar plates (bioMérieux SA, Marcy l’Etoile, France) and subsequent suspensions were adjusted to an optical density of 1 McF at 900 nm using a bioMérieux ATB 1550 densitometer and streaked on the surface of Brucella Agar plates. Etests (bioMérieux SA, Marcy l’Etoile, France) were placed on each plate and these contained the following antibiotic gradients: clindamycin (CL), erythromycin (EM), ciprofloxacin (CI), moxifloxacin (MX), metronidazole (MTZ) and vancomycin (VA). The plates were incubated under anaerobic conditions at 37 °C for 48 h, according to the manufacturer’s instructions. The Clinical and Laboratory Standards Institute (CLSI) recommendations were used to define antibiotic resistance [18]. Quality control strains (*Bacteroides fragilis* NCTC 11295, *Bacteroides thetaiotaomicron* ATCC 29741, *Escherichia coli* ATCC 25922 and *Staphylococcus aureus* ATCC 25923) were included in all experiments.

#### Environmental decontamination and extended cleaning programme

Chlorine bleach was used to clean the sanitary facilities in rooms occupied by patients diagnosed with CDI. Indomethacin and Octenisept were used to clean all medical equipment and furniture. Sanitary ware and hand-contact surfaces were cleaned on a daily basis until post-outbreak to maintain high standards of hygiene. An additional terminal clean with the hydrogen peroxide vapour (HPV) decontamination technology was completed to eliminate any spores left behind in the environment. Adjacent rooms received a full final cleansing upon discharge of CDI-positive patients.

## Results

### Epidemiological and clinical findings

CDI was observed sporadically among patients hospitalised in the nephrology unit between January 2012 and October 2012. Only seven cases of CDI were confirmed during this period. Between 26 November 2012 and 17 December 2012, an increased incidence of CDI was observed among dialysis patients hospitalised in the nephrology unit. The demographic and clinical data are presented in Table 1. The outbreak involved nine patients (five women and four men) in total, whose ages ranged from 52 to 89 years (with a median age of 70.5 years). Watery diarrhoea was observed in all patients, and was the major clinical manifestation of CDI. Three patients developed recurrent CDI. Two patients died of complications due to CDI, yielding a mortality rate of 22 % (2/9).

### History of outbreak

**Fig. 1:**
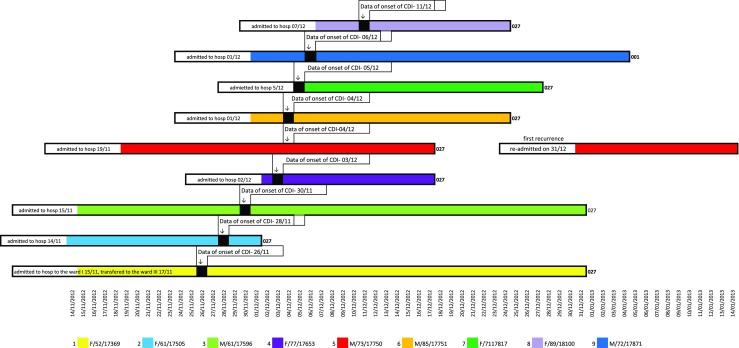
A history of symptoms of CDI among all dialysis patients hospitalised in the nephrology unit

Initially, two cases of CDI were detected in the nephrology unit. The first patient was admitted to the internal department of cardiovascular diabetology on 15 November. Subsequently, the patient was transferred to the nephrology unit. Since his risk of acquiring *C. difficile* was high, he was placed under isolation. CDI was diagnosed on 26 November. Patient 2 was admitted on 14 November. CDI was diagnosed after 14 days of hospitalisation. A history of the symptoms of CDI among all dialysis patients hospitalised in the nephrology unit is shown in Fig. 1.

### Microbiological analysis

Of the nine *C. difficile* isolates, eight contained the *tcdB* gene, the binary toxin genes (*cdtA* and *cdtB*) and a deletion in the *tcdC* gene, and one strain possessed the *tcdB* gene. All eight isolates positive binary toxin genes were recognised by the Xpert assay as belonging to the PCR ribotype 027 strain of *C. difficile*. PCR ribotyping classified the nine *C. difficile* isolates into two ribotypes: 027 (*n* = 8) and 001 (*n* = 1). Susceptibility testing was performed for all strains. All nine strains were found to be resistant to CI [minimum inhibitory concentration (MIC) ≥ 32 mg/L], while the eight PCR ribotype 027 strains were resistant to MX (MIC ≥ 32 mg/L). Only type 001 was susceptible to all the other antibiotics. The MIC of CL was 0.38–6 mg/L, while that of EM was 0.75–256 mg/L. All PCR ribotype 027 strains were resistant to EM. Nine strains were susceptible to MTZ (MIC range 0.094–0.38 mg/L) and VA (MIC range 0.25–0.5 mg/L).

## Discussion

We describe the first outbreak of CDI caused by the *C. difficile* PCR ribotype 027 strain in dialysis patients in Poland. In an earlier study, 175 *C. difficile* strains were isolated from patients hospitalised in a teaching hospital in Warsaw during 2005–2006. Of these, only one isolate belonged to PCR ribotype 027 [19]. This ribotype was not observed in Poland until 2008. In the period 2008–2010, outbreaks of antibiotic-associated diarrhoea occurred in three different hospitals in Poland [20]. We concluded that outbreaks of CDI associated with hypervirulent strains belonging to PCR ribotype 027 occurred in hospitals in Poland in 2008–2010.

All patients with diarrhoea were promptly identified and isolated. Strict isolation procedures remained in place for 30 days after the last identified case. All symptomatic patients remained in isolation with dedicated toilets and shower facilities until discharge, regardless of symptoms. In an effort to reduce the incidence of CDI, the hospital used accelerated hydrogen peroxide as the hospital disinfectant. Accelerated hydrogen peroxide has been shown to inactivate *C difficile* spores within a 15-min window [21, 22]. This ward was then disinfected with non-buffered hypochlorite, after which the outbreak ended. Fawley et al. compared the effects of five different cleaning agents on epidemic and non-epidemic *C. difficile* strains [23]. Their study showed that only chlorine-based germicides were able to inactivate *C. difficile* spores. Martinez et al. support the use of chlorine-based disinfectants for preventing the transmission of *C. difficile* [24]. The implementation of strict infection-control precautions, antimicrobial stewardship and enhanced environmental cleaning are key components in managing a *C. difficile* outbreak successfully. The outbreak at PH was controlled successfully by terminal cleaning of the ward and reinforcement of infection-control precautions.

Our findings suggest that dialysis patients who are treated with antimicrobial agents are at a risk of developing CDI. The outbreak at PH was controlled successfully. Our report emphasises the importance of monitoring CDIs and the early identification of symptomatic patients to prevent the spread of infection.
